# Global Trends in Research of Androgen Receptor Associated With Breast Cancer From 2011 to 2020: A Scientometric Analysis

**DOI:** 10.3389/fendo.2022.887612

**Published:** 2022-06-21

**Authors:** Lingzhi Chen, Yiyuan Liu, Jiehui Cai, Zeqi Ji, Juan Zou, Yaokun Chen, Jinyao Wu, Daitian Zheng, Jiehua Zheng, Yexi Chen, Zhiyang Li

**Affiliations:** ^1^ Department of Thyroid, Breast and Hernia Surgery, The Second Affiliated Hospital of Shantou University Medical College, Shantou, China; ^2^ Department of Breast Disease Research Center, Medical Research Institute of Shantou Doctoral Association, Shantou, China

**Keywords:** breast cancer, androgen receptor, scientometrics, bibliometrix, VOSviewer, CiteSpace

## Abstract

Recently, the androgen receptor has been found as a potential prognostic index and therapeutic target for breast cancer. To reveal the current research status and hotspots in this area, we analyzed the characteristics of related publications from 2011 to 2020. All related publications from 2011 to 2020 were retrieved from the Web of Science. Biblioshiny, VOSviewer, and CiteSpace V were applied to obtain the information on annual publications and citations, the highest yielding countries and authors, influential journals and articles, as well as hot keywords. In total, 2,118 documents, including 1,584 original articles and 534 reviews, were retrieved. Annual publication output was rich from 2014 to 2018, reaching the top in 2017. A systematic review written by Lehman et al. in 2011 was the most-cited document and reference. The United States was the leading country with the maximum number of publications, citations, and link strengths with other countries. The journal publishing the most was *Oncotarget*. Lehmann was the author who had the highest link strengths with other authors. The most highlighted keywords were “androgen receptor” (*n* = 1,209), “breast cancer” (*n* = 690), “expression” (*n* = 545), “breast cancer” (*n* = 410), “prostate cancer” (*n* = 290), and so on, revealing the trend from molecular mechanism level to therapeutic use level. The androgen receptor plays a significant role in the development of breast cancers, whereas its therapeutic value seems to be controversial and needs further study. With the help of a scientometric analysis in this field, researchers can clarify the current research status and hotspots worth fully exploring.

## Introduction

Before 2020, lung cancer was the leading cancer of incidence for women in the world. Nevertheless, according to the latest global cancer statistics in 2020, breast cancer has officially surpassed lung cancer and become the most common malignancy affecting women throughout the world (1). In addition, breast cancer keeps the first place in cancer mortality in women globally in 2020 (2). In China, breast cancer is the most common cancer, representing approximately 18% of the global incidence and taking the place of liver cancer as the fourth cause of death, following lung cancer, colorectal cancer, and gastric cancer (1, 3). In view of the increasing burden of breast cancer, it is urgent to develop an effective and accurate treatment to overcome this medical problem worldwide.

It is well understood that androgen receptors (ARs) play an important role in prostate cancer (4). AR-targeted therapies are effectively applied in prostate cancer (5–7). Like prostate cancer, breast cancer is associated with sex hormones such as estrogen and progesterone (8, 9). The use of aromatase inhibitors and selected estrogen receptor modulators is globally recognized in blocking the estrogen signaling pathways (10–13). However, not all types of breast cancers are responsive to estrogen-targeted therapy, and the emergence of endocrine therapy resistance is tricky (14, 15). For example, triple-negative breast cancers, where the expression of estrogen receptor, progestogen receptor, and human epidermal growth factor receptor 2-related genes is absent, do not benefit from current targeted therapies (16).

Nowadays, many studies have shown that ARs may be a new potential prognostic index and therapeutic target for some types of breast cancer when other therapeutic methods are insufficient to inhibit tumor progression (17–22). There are some reviews that have summarized the physiology of ARs, the mechanisms involved, and AR-targeted therapies in breast cancers (23–26). Although related studies have been completed or are ongoing, it seems that ARs have not been taken seriously in clinical practice.

Given this situation, we conducted a scientometric analysis of relevant articles in this field so that researchers could clarify the current research status. The purpose of our analysis is to obtain information on annual publications and citations in related fields, the highest yielding countries and authors, influential journals, hot topics, and keyword analysis in the past decade, providing researchers with potential future research directions.

## Materials and Methods

### Data Collection

We retrieved all publications related to the relationship between androgen receptors and breast cancer from Clarivate analysis’s Web of Science Core collection database, including editions of SCI-EXPANDED (2003–present), SSCI (2003–present), A&HCI (2003–present), ESCI (2015–present), CCR-EXPANDED (1985–present), and IC (1993–present). The medical subject headings (Mesh) and entry terms “androgen receptor” and “breast cancer” were used as search strategies. The search query includes the following: #1, ALL=(“androgen receptor*”) OR ALL=(“dihydrotestosterone receptor*”) OR ALL=(“testosterone receptor*”) OR ALL=(“stanolone receptor*”) OR ALL=(“5 alpha dihydrotestosterone receptor*”); #2, ALL=(“breast neoplasm*”) OR ALL=(“breast tumor*”) OR ALL=(“breast cancer*”) OR ALL=(“breast carcinoma*”) OR ALL=(“mammary cancer*”) OR ALL=(“mammary carcinoma*”) OR ALL=(“mammary neoplasm*”); #3, “#1”, and “#2”. Also, the timespan of these publications was then filtered from 2011 to 2020. The research was conducted on October 3, 2021, and produced a total of 2,514 documents. We then set the document types to articles or reviews, restricted the language to English, and got a result of 2,134 publications, including 1,600 articles and 534 review articles. In total, 380 publications were excluded: 5 non-English articles, 2 non-English review articles, 340 meeting abstracts, 17 editorial materials, 11 letters, 3 corrections, 1 retracted publication, and 1 reprint. Using the Zotero software to manage the collected literature, we found 3 more retracted articles and 13 articles published in 2021 and then excluded them. Lastly, the number of publications included in the scientometric analysis was 2,118, including 1,584 articles, which accounted for 74.79% of the whole, and 534 review articles (25.21%).

### Data Analysis

The online “analyze results” function in the Web of Science (WoS) was preliminarily applied to gain information about the publication years of these publications, document types, research areas, authors, affiliations, journals, publishers, countries, languages, funding agencies, and open access. Other information such as the number of all citing articles and citing articles without self-citations, the sum of times cited and times cited without self-citations, average citations per term (ACI), and h-index was also acquired from the “create citation report” function of the WoS.

The information from these publications we collected, including titles, authors, affiliations, languages, document types, abstracts, keywords, and cited references, was imported into Biblioshiny (a webinterface for Bibliometrix), VOSviewer software, and CiteSpace software.

Bibliometrix, an R-tool of R-studio (version 4.1.0), was used for the complete scientometric analysis of the data from the WoS and to produce images to visualize the results (27). Importing the raw files of the retrieved data into the Biblioshiny website, we acquired the main information of these publications, including timespan, quantity of sources, quantity of documents, quantity of references, document types, document contents, authors, and authors’ collaboration. With the information, we reached a preliminary judgment on whether the results met the inclusive criteria. Moreover, other information included annual scientific production, average article citations per year, sources (most relevant sources, journal sources, and source dynamics), authors (most relevant authors, authors’ production overtime, author impact ranked by h-index, g-index, and m-index), countries’ and affiliations’ contribution, most cited documents and references, and keywords. Relationships between the most productive countries, the top authors, and affiliations were summarized by a three-field plot. The co-occurrence network of keyword plus was the measure aiding in detecting the hot research focus.

VOSviewer (version 1.6.17), as a network analysis software, was applied to analyze the most cited documents and construct a co-citation network analysis of references and a network analysis of keyword co-occurrence. Also, the link strength among different countries, affiliations, and authors in our study was also acquired from this software (28).

CiteSpace V (version 5.8.R3) contributed to getting citation bursts for references and finding the keywords with strong citation bursts (29).

Statistical analyses were conducted with IBM SPSS statistics 26. We selected the numerical variables such as the quantity of annual publication output and represented them as means with standard deviation and medians with maximum and minimum. The categorical variables were expressed as frequency or percentage. The Spearman correlation coefficient was used to find if there were some correlations between selected continuous variables and verify the statistical significance. All tests were two-sided, and a *p*-value of less than 0.05 was regarded as statistically significant.

**Figure 1:**
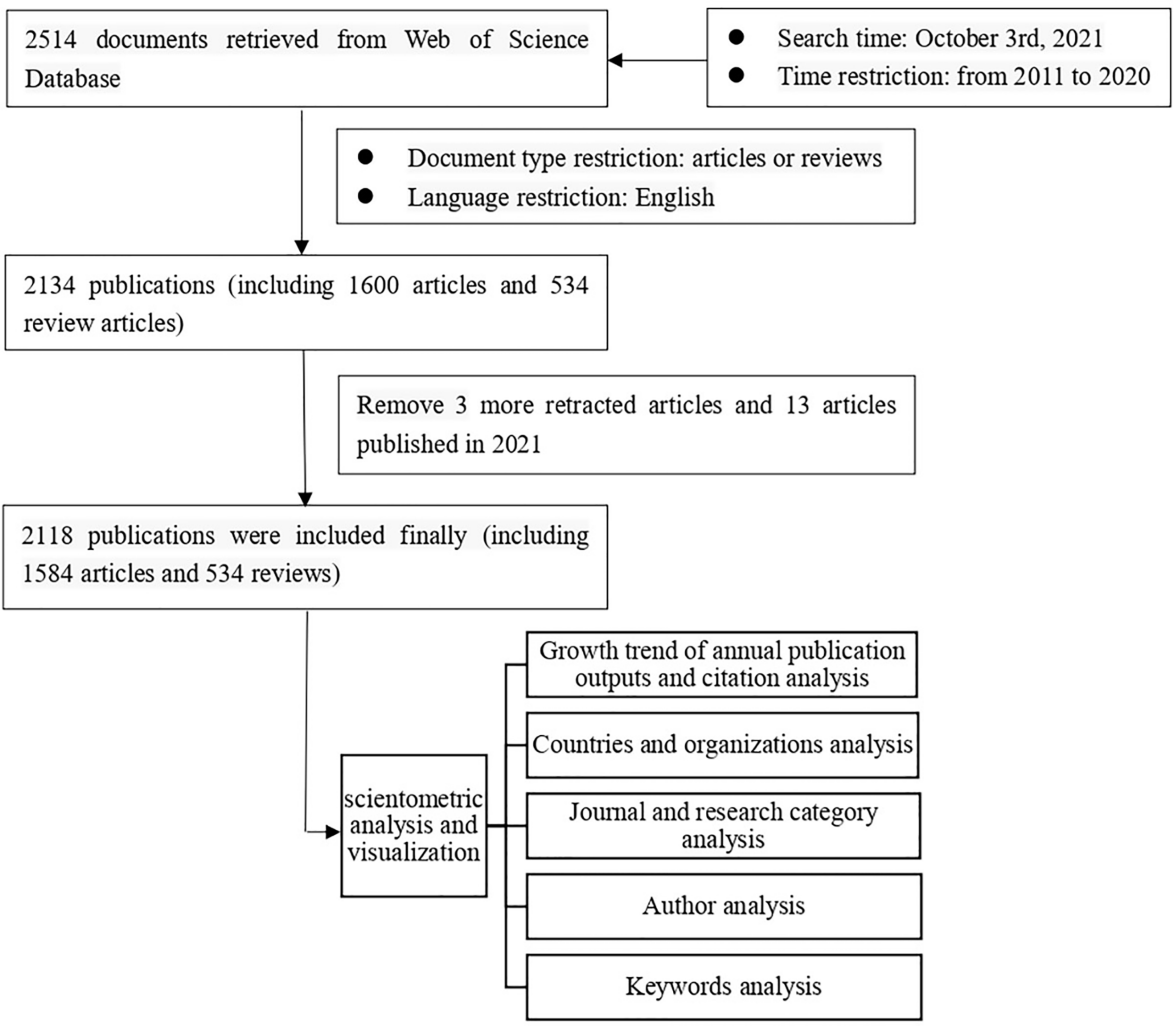
Flowchart of data collection and analysis.

**Figure 1** shows the step taken to collect the literature and analyze the results. This study did not require the approval of the ethics committee.

## Results

### Annual Publication Output Growth Trend and Citation Analysis

**Figure 2:**
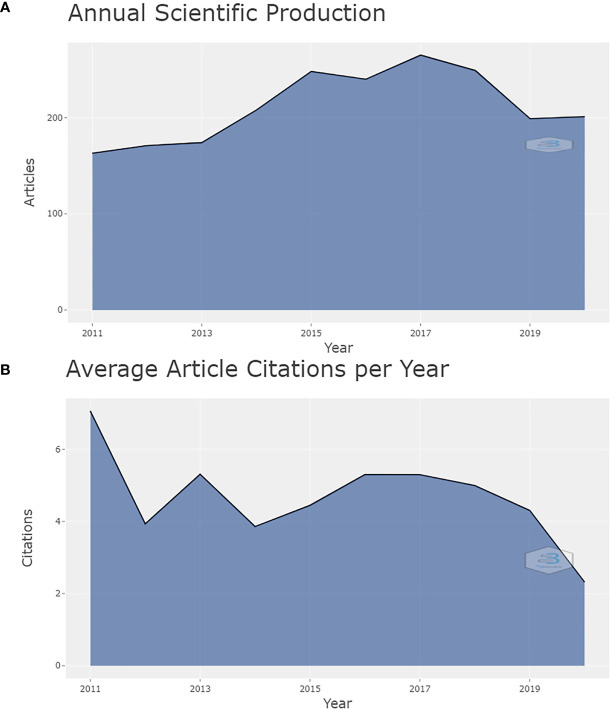
Annual scientific production and average article citations per year from 2011 to 2020 in this research field. **(A)** Annual scientific production from 2011 to 2020 in this research field. **(B)** Average article citations per year from 2011 to 2020 in this research field.

The total number of documents related to this topic in the last 10 years we retrieved from the WoS was 2,118; 2017 is the year contributing the most quantity of publication output (265, 12.51%). It displayed an upward trend from 163 documents (7.70%) published in 2011 to 265 documents (12.51%) published in 2017, while the overall trend seems to be stable between 2011 and 2020, as shown in **Figure 2A** (*r* ^2^ = 0.539 [CI, −0.061 to 0.964]; *p* = 0.108]). The period from 2014 to 2018 was a rapid development period. It was noteworthy that the publication’s output per year had a sudden increase (growth rate of 19.54%) and broke through 200 in 2014. The median annual growth rate of the increasing annual scientific production was 7.67%, with a maximum in 2014 (19.54%) and a minimum in 2020 (1.01%). From 2018, the publication’s output started to decrease, but it increased a little in 2020. The lowest growth rate was recorded in 2019 (−20.08%). Citation analysis is a simple and objective way to evaluate the quality of published articles, journals, research organizations, and even individual researchers. The number of times an article is cited reflects its scientific impact (30). Among all the retrieved articles, there were 44,316 citing documents, including 1,355 self-citations and 42,961 without self-citation (accounting for 96.94% of all citing documents). The sum of the number of times an article was cited was 65,051, including 56,696 without self-citation, which accounted for 87.16%. This showed a rising trend over the years from 189 in 2011 to 12,219 in 2020. (*r* ^2^ = 0.994 [CI, 0.991 to 1.000]; *p* < 0.001]). The average time cited per document was 30.47. **Figure 2B** shows the average article citations per year from 2011 to 2020, with the most in 2011 (7.06), delineating a fluctuating trend from 2011 to 2016 and a declining trend since 2016.

We use VOSviewer to view **Supplementary Table S1**, which shows the details of the top 10-cited documents in the research scope of androgen receptors and breast cancer. The highly cited paper indicated the frontier research area for us. These studies were all published between 2011 and 2017. Around 60% of them were published in 2011; even the most cited article was published in 2011. The most cited paper (Lehman et al., 2011), which was cited 2,766 times, is a systematic review (16). The work “Triple-negative breast cancer: challenges and opportunities of a heterogeneous disease” by Bianchini et al. (2016) received 926 immediate citations (31). The third-ranked article was “Pioneer transcription factors: establishing competence for gene expression” written by Zaret and Carroll (2011) (cited 924 times) (32). There were 6 studies cited over 500 times, which occupied 0.28% of all citations; 16 (0.76%) documents were cited over 300 times and 100 (4.72%) over 100 times. In the last 5 years, the most cited paper (Lehman et al., 2011) was cited 1,697 times (61.35% of all its cited times) and the ranked No. 2 cited article (Bianchini, et al., 2016) was cited 921 times (99.46% of all its cited times).

**Figure 3:**
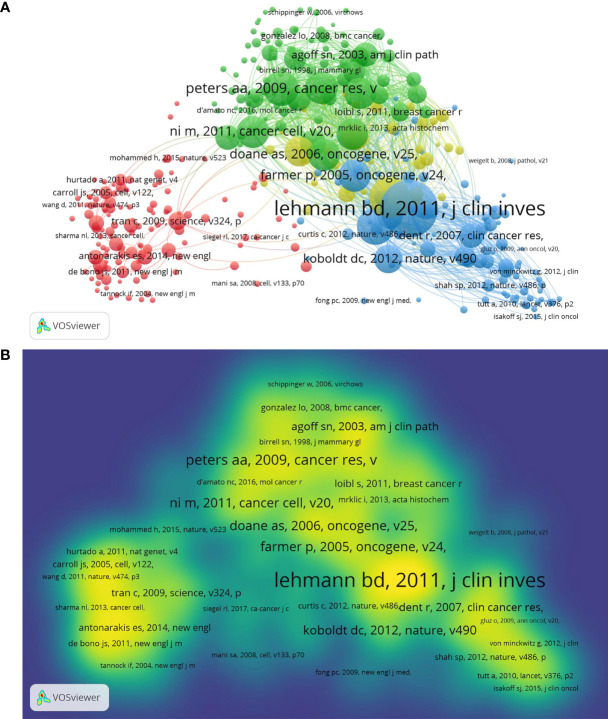
Co-cited network mapping of cited references of these documents. **(A)** Network map of co-citation between references with more than 20 citations. **(B)** Density map of co-citation between references with more than 20 citations.

**Figure 3** represents a co-cited network diagram of the references of these documents conducted by VOSviewer. Using 20 as the minimum number of citations of a cited reference, 356 references met the threshold. The more references cited, the larger the node (**Figure 3A**). A yellower color means more co-citations (**Figure 3B**). There are four clusters of the cited references in **Figure 3A**. The top cluster with 124 items suggests the most attractive research field, which is shown in red. In **Figure 3**, the article written by Lehmann (2011) had the most citations of 333, representing that this reference counted for a great deal (16). The top five references with the most citations were Lehmann (2011) (333 citations) (16), Gucalp (2013) (226 citations) (33), Perou (2000) (204 citations) (34), Peters (2009) (192 citations) (35), and Hu (2011) (178 citations) (36). Using CiteSpace, we detected the top 25 references with the strongest citation bursts (**Supplementary Figure S1**). Notably, 40% of them (10/25) developed citation bursts in 2011, followed by 2018 (6/25, 24%) and 2012 (4/25, 16%). In addition, 7 references (28%) were continuously cited until 2020. The reference with the strongest burstness (strength = 33.18) was also the paper written by Lehmann in 2011, with citation bursts from 2013 to 2016 (16).

### Analysis of Countries and Organizations

**Figure 4:**
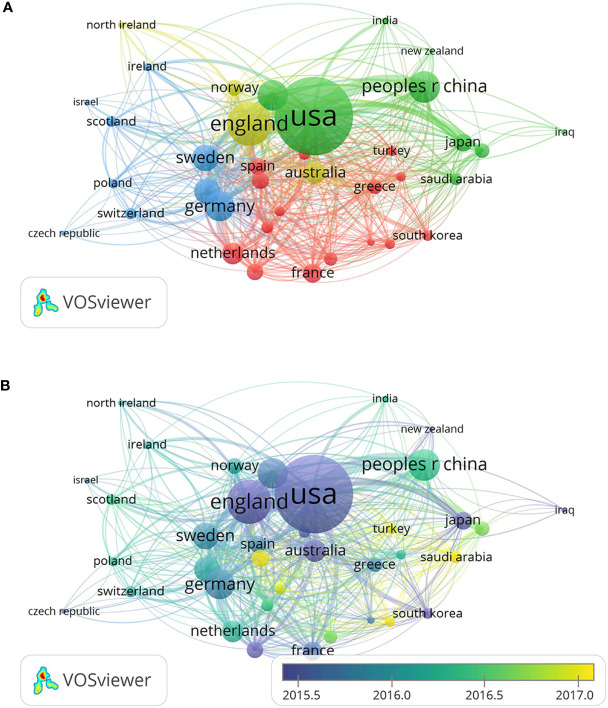
Co-authorship analysis of countries in this field. **(A)** Network map of co-authorship between countries with more than five publications. **(B)** Overlay map of co-authorship between countries with more than five publications. (The blue color represents the earlier years; the yellow color represents the more recent years.).

Of these 2,118 documents, 85 countries and regions contributed to this research field. They are developed countries and developing countries as well. The United States was the most productive country which published the maximum number of related studies (*n* = 878, accounting for 41.45% of all). It was followed by China (*n* = 321, 15.16%), Japan (*n* = 161, 7.60%), Italy (*n* = 142, 6.70%), and England (*n* = 136, 6.42%). The top 10-cited countries are shown in **Table 1**. The publications from the United States had the most citations (*n* = 31,363), and then China ranked second (*n* = 5,036) and was followed by Australia (*n* = 3,593), Italy (*n* = 3,171), and England (*n* = 3,136). In co-authorship analysis, a total of 52 countries with more than 5 publications in this research area were analyzed. Sequenced by the total link strength, the top 5 countries were the United States (total link strength = 554), England (262), China (186), Canada (168), and Germany (162) (**Figure 4**). The size of the circle reflects the total link strength of different countries, and the distance between circles represents the strength of their relationship according to their frequency of co-occurrences. As time went by, some developing countries began to cut a striking figure in this area, for example, China (total link strength = 186), Egypt (total link strength = 67), Hungary (total link strength = 45), and India (total link strength = 30).

**Table 1:** Top 10 cited countries contributing to this research area.

Country	Total production	Total citations	Average article citations
USA	878	31,363	44.30
China	321	5036	17.86
Australia	96	3,593	47.28
Italy	142	3,171	29.09
UK	136	3,136	37.78
Japan	161	2,877	21.31
France	74	1,801	35.31
Germany	106	1,707	26.26
Canada	117	1,648	21.13
Korea	85	1,528	20.37

**Figure 5:**
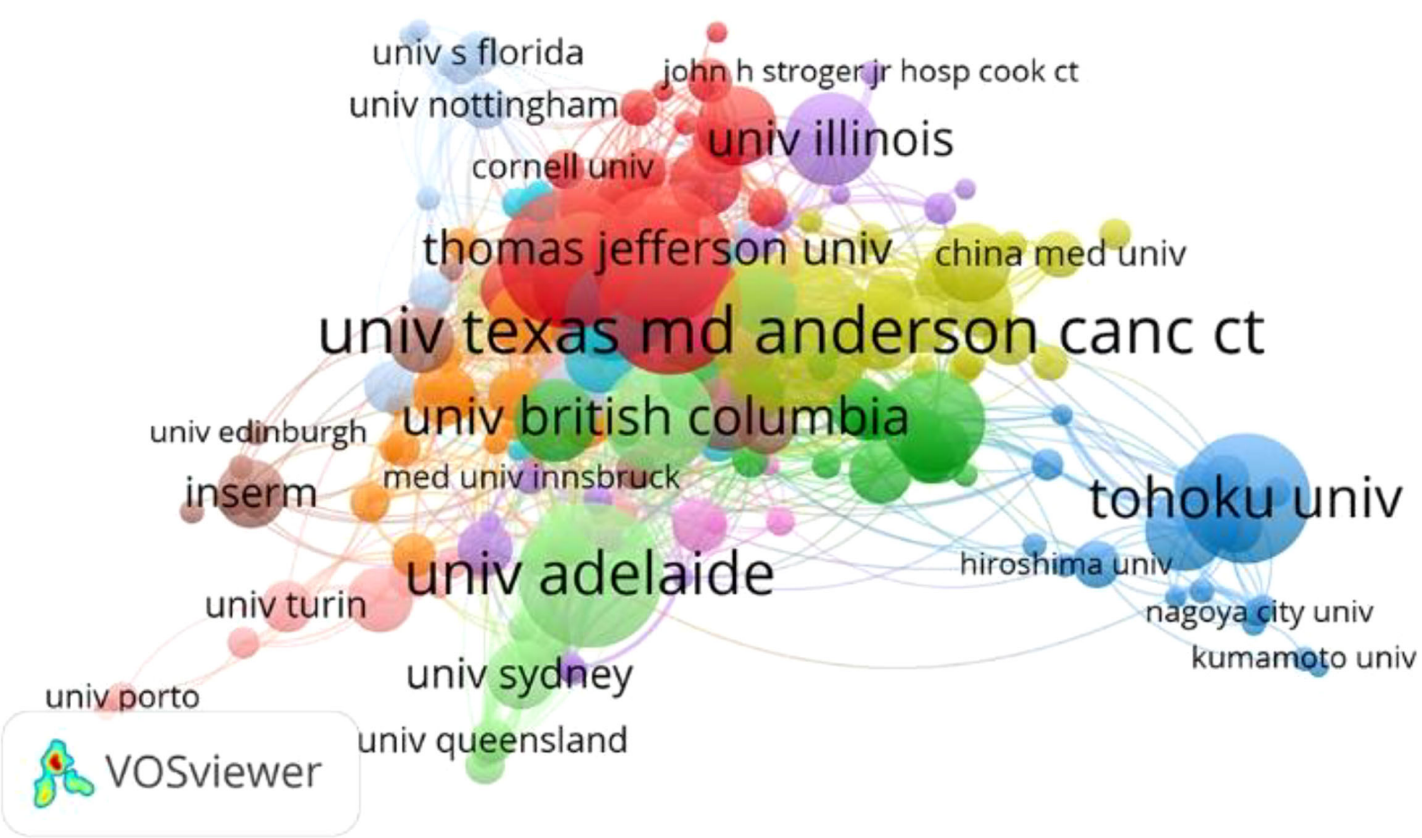
Network map of co-authorship between organizations in this field.

A total of 2,687 institutions have been involved in this field. The University of Texas MD Anderson Cancer Center contributed the maximum number of publications (*n* = 47, 2.22%) and was followed by the University of Michigan (*n* = 41, 1.94%), the University of Adelaide (*n* = 41, 1.94%), the Memorial Sloan Kettering Cancer Center (*n* = 40, 1.89%), and Baylor College of Medicine (*n* = 39, 1.84%) (**Table 2**; **Figure 5**). We then analyzed the co-authorship of organizations with more than 5 publications and got a result of 260 organizations. The top five organizations with the total link strength were the University of Michigan (123), the University of Cambridge (113), the University of Washington (111), Harvard University (109), and the Dana-Farber Cancer Institute (105).

**Table 2:** Most productive organizations contributing to this research field.

Organization	Total production	Total citations	Total link strength
The University of Texas MD Anderson Cancer Center	47	3,487	73
University of Michigan	41	1,521	123
University of Adelaide	41	1,706	64
Memorial Sloan Kettering Cancer Center	40	2,121	88
Baylor College of Medicine	39	1,720	50
Tohoku University	36	832	24
University of British Columbia	31	1,266	103
Harvard University	29	2,045	109
Tianjin Medical University	28	569	17
Dana-Farber Cancer Institute	27	2,002	105

### Journal and Research Category Analysis

These 2,118 documents were published in 614 kinds of journals. The top 10 most productive journals in this area are shown in **Supplementary Table S2**. These top 10 journals published a total of 444 documents, accounting for 20.96% of all. *Oncotarget* published the most document (*n* = 72). The second most popular journal was *PLOS One*, which has 63 publications. *Breast Cancer Research and Treatment* ranked No. 3 (*n* = 50) and was followed by *Endocrine-Related Cancer* (*n* = 44), *Cancers* (*n* = 40), and *Clinical Cancer Research* (*n* = 40). These journals mainly focus on oncology. *Clinical Cancer Research* had the most citations with 3,077 and was followed by *Journal of Clinical Investigation* (3,067 citations), *Nature Reviews Cancer* (2,464 citations), *Oncogene* (2,087 citations), and *Oncotarget* (1,841 citations). With regard to source impact, the h-index was used to describe these journals’ importance (37). Both *Oncotarget* and *Clinical Cancer Research* have the largest h-index of 27, followed by *Oncogene* (h-index = 25), *PLOS One* (h-index = 24), and *Endocrine-Related Cancer* (h-index = 24) (**Table 3**). *Oncotarget*, an open-access impact journal, started publishing in 2009 but is not currently indexed for Medline. Over the years, *PLOS One*, *Clinical Cancer Research*, *Breast Cancer Research and Treatment*, and *Endocrine-Related Cancer* have stayed active in this field. *Oncotarget* became active 5 years ago but has shown a decrease in recent years while *Cancers* became active in the last 2 years (**Supplementary Figure S2**).

**Table 3:** Source impact of the top 10 journals publishing in this area.

Source	h-index	g-index	m-index	TC	NP	PY start
Clinical Cancer Research	27	40	2.454545455	3,077	40	2011
Oncotarget	27	38	3	1,841	72	2013
Oncogene	25	33	2.272727273	2,087	33	2011
Endocrine-Related Cancer	24	35	2.181818182	1,324	44	2011
PLOS One	24	39	2.181818182	1,725	60	2011
Breast Cancer Research and Treatment	21	34	1.909090909	1,275	47	2011
Cancer Research	21	22	1.909090909	948	22	2011
Cancers	19	26	2.375	725	38	2014
Molecular and Cellular Endocrinology	17	25	1.545454545	661	32	2011
BMC Cancer	16	26	1.454545455	737	35	2011

In terms of research categories, a total of 81 research categories were involved. Based on the quantity of publications, the top 5 subject categories are listed in **Table 4**: “Oncology” (967 publications), “Cell Biology” (308 publications), “Biochemistry Molecular Biology” (301 publications), “Endocrinology Metabolism” (286 publications), and “Pathology” (170 publications). As for the publishers, the most represented publishers based on the number of publications were “Springer Nature” (436 publications), “Elsevier” (383 publications), “Wiley” (177 publications), “American Association Cancer Research” (97 publications), and “MDPI” (84 publications).

**Table 4:** Top 5 active research subject categories.

Research subject category	Documents
Oncology	967
Cell biology	308
Biochemistry molecular biology	301
Endocrinology metabolism	286
Pathology	170

### Author Analysis

In our dataset, 11,950 authors contributed to these 2,118 publications, including 46 authors of single-authored documents and 11,904 authors of multiauthored documents. The total number of authors’ appearances was 16,359, while the total number of authors was 11,950. There were 53 single-authored documents. The average number of authors per document was 5.64, and the average number of co-authors per document was 7.72. Tilley was the most productive author and he contributed to 31 publications, accounting for 1.46% of all, followed by Sasano with 29 publications (1.37%) and then Hickey (*n* = 21, 0.99%), McNamara (*n* = 20, 0.94%), and Brown (*n* = 19, 0.90%) (**Table 5**). The more productive authors were more central and important in this field. Four authors have authored over 20 articles, and 36 have authored over 10 articles. A total of 6,192 authors (51.82%) have just begun to publish related articles in the last 5 years. In **Supplementary Figure S3**, the dot size relates to the number of publications and the shade of the color in proportion to the total number of citations per year. Tilley’s and Hickey’s articles were mainly published in the first 5 years, while Sasano and McNamara kept a steady trend generally. Brown performed actively in the first 2 years and presented ups and downs in the last 5 years.

**Table 5:** Top 10 contributing authors in the field of androgen receptor and breast cancer.

Authors	Articles	Articles fractionalized	h-index	g-index	m-index	TC	NP	PY_start
Tilley WD	31	4.39	21	30	1.750	1,314	30	2011
Sasano H	29	4.64	13	24	1.182	608	29	2012
Hickey TE	21	3.02	14	20	1.167	862	20	2011
McNamara KM	20	3.55	11	20	1.100	456	20	2013
Brown M	19	2.28	14	19	1.167	1,187	19	2011
Niu Y	18	2.82	9	14	0.750	216	17	2011
Suzuki T	18	1.91	12	18	1.000	514	18	2011
Inoue S	17	3.83	15	17	1.250	506	17	2011
Li Y	17	2.13	13	17	1.182	420	17	2012
Miki Y	17	1.91	11	17	1.000	348	17	2012

In terms of the times cited in their publications, the top 5 authors are shown in **Supplementary Table S3**. Sanders ranked No. 1 with 3,879 citations, followed by Lehman and Pietenpol (3,495 citations), Chen (3,442 citations), Bauer (2,929 citations), and Shyr (2,790 citations). Based on the h-index, Tilley was the top 1 (h-index = 21), Inoue ranked No. 2 (h-index = 15), and Brown (h-index = 14) and Hickey (h-index = 14) ranked No. 3, respectively, and they were followed by Carroll, Li, and Sasano (h-index = 13) and Selth, Suzuki, and Wang (h-index = 12). Among the most productive authors, there were 15 with an h-index of over 10. Taking scientists of different seniority into account, the m-index serves as a remedy for correcting the h-index for time, helping to identify truly successful scientists (37). Tilley has the highest m-index of 1.750 and then Guan (m-index = 1.286), Inoue (m-index = 1.250), Li and Sasano (m-index = 1.182), and Brown (m-index = 1.167) followed him. A total of 171 authors with over 5 publications were analyzed. The top 5 authors with the highest link strength were Sasano (total link strength = 106), Suzuki (total link strength = 87), Tilley (total link strength = 83), Ishida (total link strength = 78), and Miki (total link strength = 76).

**Figure 6:**
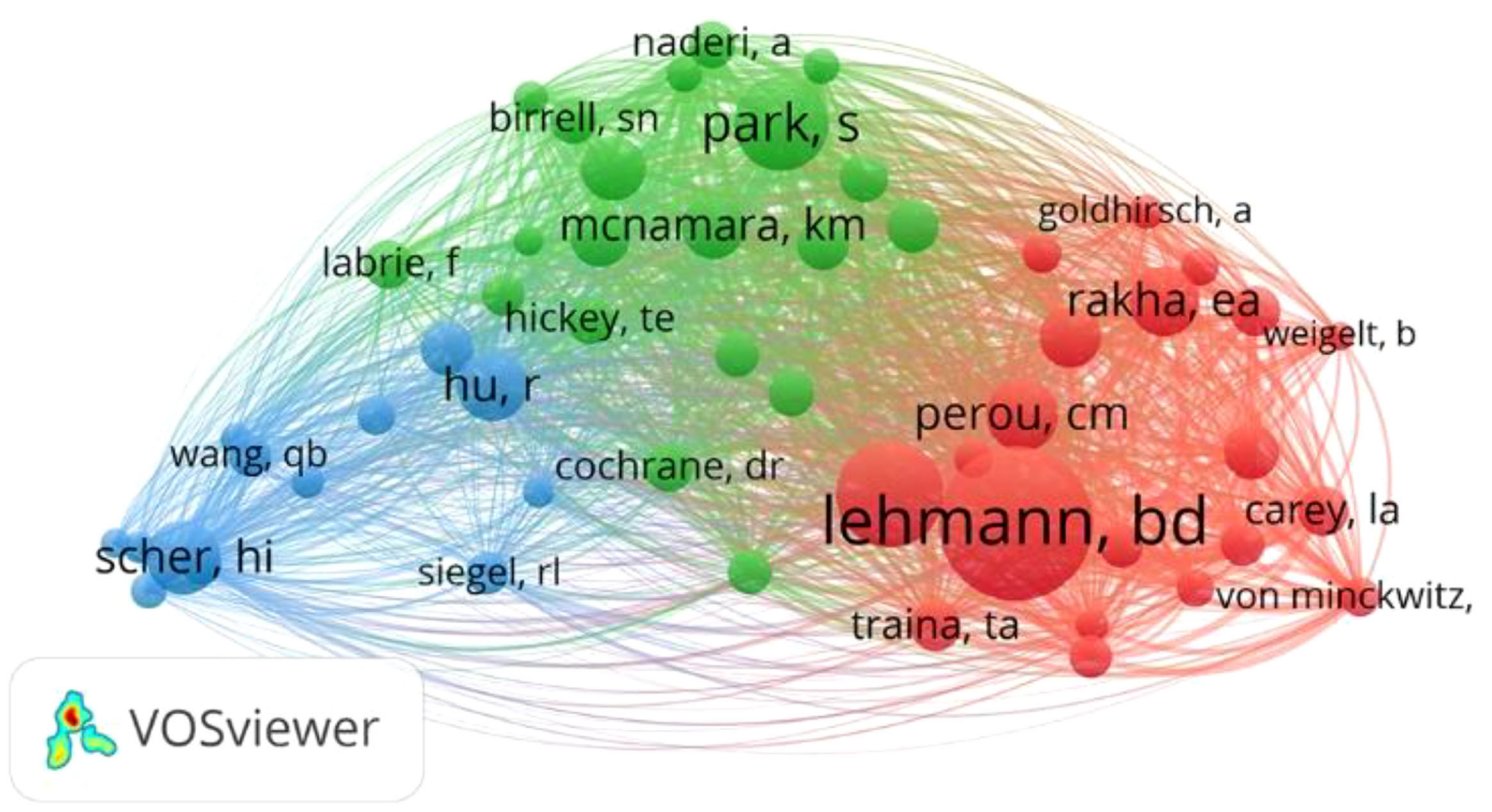
Network map of co-citations between authors with the minimum citations over 100 times.

We use VOSviewer software to perform co-citation analysis of authors (**Figure 6**). Fifty-three authors were analyzed with a minimum of citations of over 100 times. The top 5 authors with the highest link strength in co-citation analysis were Lehmann (total link strength = 6,164), Gucalp (total link strength = 4,587), Park (total link strength = 3,764), Perou (total link strength = 2,799), and Rakha (total link strength = 2,798).

The three-field plot (**Supplementary Figure S4**) presented the relationships among institutions (right), the most productive countries (middle), and top authors (left). The height of nodes and the thickness of lines represent the contribution and the number of connections, respectively, of a country, author, and institution. The United States was the country with the most connections (*n* = 35), followed by China (*n* = 34) and Japan (*n* = 28). The top contributing institution in the United States was the University of Michigan (17/35 = 48.57%), followed by the Memorial Sloan Kettering Cancer Center (16/35 = 45.71%). The top 3 authors with the strongest collaboration trend among different countries were Kim, Brown, and Tilley.

### Analysis of Keywords

A total of 815 keywords with more than 5 occurrences were analyzed by VOSviewer. We use the clustering function in VOSviewer to divide the whole co-occurrence network into several clusters. Keywords with higher correlations are more likely to be put into the same cluster with the same color. As shown in the network visualization map (**Supplementary Figure S7A**), all these selected keywords could be roughly divided into 10 clusters. This helps to find the focus of recent research. The density visualization map performed the keywords with the same frequency (**Supplementary Figure S7C**). The most frequently used keywords were “androgen receptor” (*n* = 1,209), “breast-cancer” (*n* = 690), “expression” (*n* = 545), “breast cancer” (*n* = 410), “prostate-cancer” (*n* = 290), and so on. Additionally, the color of the overlay visualization map indicates the average time when these keywords occurred in their articles (**Supplementary Figure S7B**). The more yellow the color is, the later the keyword occurs. As time went by, the main emphasis of the research area shifted from the relationship between the molecular types of breast cancer and AR gene expression to the mechanism and signal pathway of AR’s action in breast cancer and then to the clinical application such as therapy and prognosis.

Keywords plus are the words or phrases that frequently appear in the titles of the references cited by the retrieved article but not in the title of this retrieved article (38). **Supplementary Figure S5** is composed of four quadrants in which the words or phrases have different properties: (a) Motor themes (first quadrant): words in this cluster have high centrality and high density thus they are well developed and important for this research field. (b) Highly developed and isolated themes (second quadrant): they have high density and low centrality, meaning that they have been well developed but are not much important now because of their lack of links with other themes. (c) Emerging or declining themes (third quadrant): they have low centrality and low density. These themes have poor development and relationships in this research area. Maybe they are just emerging, or maybe they are disappearing. (d) Basic themes (fourth quadrant): they have high centrality but low density. They are the basic concepts and knowledge in this research field (38). These keywords plus were divided into four clusters with four colors. From **Supplementary Figure S5**, We can see cluster 1’s (red color) keywords (“androgen receptor”, “breast-cancer”, “prostate cancer”) and cluster 2’s (purple color) keywords (“expression”, “identification”, “estrogen”) were in the motor themes in this research field, while cluster 3’s (blue color) keywords (“estrogen-receptor”, “post-menopausal women”, “progesterone-receptor”) and cluster 4’s (green color) keywords (“androgen receptor expression”, “growth-factor receptor”, “neoadjuvant chemotherapy”) were in the merging or lining theme quadrant, which means they were poorly developed. Furthermore, cluster 1 (red color) and cluster 4 (green color) were in the center of the graph, which indicates that these keywords plus had a higher relevance degree in this area.

Some of these keywords were presented in references with citation bursts. The so-called burst words represent words that are cited frequently over a period. CiteSpace V was used to detect burst keywords, which are indicators of research frontier topics over time. **Supplementary Figure S6** shows the top 25 keywords with the strongest citation bursts. The first ten keywords emphasize the emerging trend of the research between breast cancer and androgen receptor in 2011–2015. The middle three keywords became active in the intermediate stage of the past 10 years. The last twelve keywords have received a lot of attention in recent years, which means they are the focus of current studies and those that will continue to be active until 2020 (“molecular subtype”, “peripheral blood”, “pathological complete response”, “tumor-infiltrating lymphocyte”, “men”, “migration”, and “small molecular inhibitor”). The keyword “pathological complete response” got hold of the strongest bursts (strength = 6.37), with citation bursts from 2018 to 2020.

## Discussion

A scientometric analysis of the publication output related to ARs and breast cancer during 2011–2020 was conducted by using Bibliometrix, VOSviewer, and CiteSpace. Up to 2020, the total number of documents on this topic was 2,118. The publication’s output was rich in the period from 2014 to 2018, reaching its peak in 2017. The median annual growth rate of publication output was 7.67% and the maximum growth rate appeared in 2014, which suggested an abrupt increase in interest in this research field, but it has decreased in recent years. Among all the retrieved articles, a total of 44,316 citations were received, with an average time cited per document of 30.47. It was found that the number of citations has increased 64-fold since 2011, reflecting the rising importance of this area; 60% of the top 10-cited papers were published in 2011. They synthesized and summarized the existing conclusions about androgen receptors along with breast cancer, showing basic knowledge of this field. Four of them are reviews predominantly on triple-negative breast cancer (16, 31, 39, 40). It is probably due to the Luminal androgen receptor (LAR) subtype of triple-negative breast cancers (TNBCs), which is abundant in AR expression. Finding a new target for TNBC is encouraging for clinicians and researchers. Among the most cited papers and references, the systematic review written by Lehman et al. in 2011 was a major landmark. One article published in “*Chemical Reviews*” describing the physiology of androgen receptors and one article on “*Genes and Development*” indicating the interaction between FOXA1 and ARs provide researchers with basic knowledge for studying ARs (32, 41). “Androgen receptor driven transcription in molecular apocrine breast cancer is mediated by FoxA1” was the article with the highest betweenness centrality, also showing a high correlation between ARs and FOXA1 in molecular apocrine breast cancer (42). Some new androgen-targeted therapies are emerging. An article published in the field of pharmacy describes a new potential drug-targeted androgen receptor. Unlike other drugs that act on the effects of ARs, it is a selective androgen receptor that degrades (43). Not much work has been done in this field currently, so it is worthy of further investigation.

We presented a total of 85 countries contributing to this research field. The United States was the leading country with the maximum number of publications, citations, and link strength with other countries. Four of the top 5 most productive institutions in this area are located in America. The organizations with the top five total link strengths are also located in America. There is no doubt that the USA had the highest contribution and collaboration with other countries and organizations. Nevertheless, the increasing contribution from some developing countries was a great encouragement to researchers in these countries.

Among the top 10 most productive journals out of 614 kinds of journals involved, 7 of them were categorized in “cancer” while the remaining 3 were respectively listed in “science” (*n* = 1), “biochemistry molecular biology” (*n* = 1), “cell biology” and “endocrinology metabolism” (*n* = 1). *Oncotarget* was once the researchers’ first consideration of publication in the last five years, but they turned their attention to *Cancers* in recent two years. *PLOS One*, *Clinical Cancer Research*, *Breast cancer research and treatment*, and *Endocrine-related Cancer* keep a good performance of publications output in this field indicating the significance of oncology, medicine, and science categories.

Tilley (*n* = 31, 1.46%), Sasano (*n* = 29, 1.37%), Hickey (*n* = 21, 0.99%), McNamara (*n* = 20, 0.94%), and Brown (*n* = 19, 0.90%) were the top 5 most productive authors in this field. Tilley also had the highest h-index and his first article related to this field was published in 2011, and there were two more correlative articles published in the same year. It is worth noting that Lehmann, who had the highest link strength with other authors, proposed 6 subtypes of TNBC (basal-like 1 (BL1), basal-like 2 (BL2), immunomodulatory (IM), mesenchymal (M), mesenchymal stem-like (MSL), Luminal androgen receptor (LAR)) in an article published in 2011, clarifying the characteristics and potential targeted therapies of 6 subtypes of triple-negative breast cancer, and then he revised them to 4 subtypes (BL1, BL2, M, and LAR) in 2016 (16, 44). His article, published in 2011, was the most cited paper, cited 2,766 times as a systematic review. He built a cornerstone for this research field, helping others to better clarify different types of cancer cells and tumor heterogeneity.

From the keyword analysis, AR gene expression in different molecular types of breast cancer was the main emphasis of the research area at the beginning and then the mechanism and signal pathway of the action of ARs took over a partial position. Recently, the main attention was shifted to clinical applications such as therapy and prognosis. The top 25 keywords with the strongest citation bursts revealed the trend from the molecular level and mechanism to the therapeutic use level, highlighting the increasing interest and need for finding a new kind of targeted drug. However, there are still many disputes about AR’s related mechanisms of action in BC, causing their uncertain prognostic relation. For example, most studies pointed out the positive effect of ARs in ER-positive BC, while in human epidermal growth factor receptor 2 (HER2)-positive BC and TNBCs, the negative effect was mostly shown (but there are still a few studies with opposite conclusions), perhaps due to tumor heterogeneity (45). It is known that ARs can interact with several key proteins and signaling pathways, such as Wnt/β-catenin, PI3K/AKT pathway, tension protein homolog (PTEN), and forkhead box protein A1 (FOXA1). In addition, the crosstalk between ARs and ERs or HER2s suggests the possible mechanism of drug resistance (21, 25, 46). Nonetheless, there are few studies to illustrate the upstream regulators of ARs. Maybe the upstream pathways of ARs can explain the discrepancy in these studies (45). From the keyword plus analysis, we can infer that the emerging themes such as “androgen receptor expression”, “growth-factor receptor”, and “neoadjuvant chemotherapy” need further investigation. Those keywords with citation bursts keeping to 2020 (“molecular subtype”, “peripheral blood”, “pathological complete response”, “tumor-infiltrating lymphocyte”, “men”, “migration”, and “small molecular inhibitor”) may be the hotspots in the next few years.

Regarding “androgen receptor expression”, we need to identify the meaningful AR threshold in breast cancer before discussing the significance of ARs. Throughout the retrieved articles, few studies have explored the cutoff point of ARs for predicting breast cancer survival. Opinions on the optimal cutoff point vary, and it has not been determined. In some studies, ROC analysis was used to determine the optimal cutoff point of AR expression. AR’s high ratio of expression (78%) provides an optimal sensitive and specific index to predict the prognosis of breast cancer, and the ratio of AR : ERα over 0.87 suggests the best outcome in ERα-positive breast cancer (*p* < 0.0001) (47). So far, there are no clinical guidelines regarding the methods to detect AR positivity. Immunohistochemistry (IHC) is the cheapest method and is widely used in the definition of AR positivity in BC. Based on expression data obtained by IHC, the differential rate of AR expression between primary and metastatic BC and the impact of multiple antibodies on AR status have explained the role of ARs or selected patients for AR-targeted therapy (47). The profile of circulating tumor cells (CTCs) in the blood, readily accessible by simple venipuncture, may be a valuable alternative for identifying AR status, especially in metastatic tumors. In addition to expressing AR proteins, AR gene expression profiles and AR phosphorylation status may be predictive factors in selecting eligible patients for AR-targeted therapy.

In addition to the therapeutic approach targeting ARs alone, the combination of AR-targeted therapy with other treatments has attracted considerable interest as a new potential strategy, such as a combination of PARP inhibitors, CDK4/6 inhibitors, PI3K inhibitors, and conventional chemotherapy with paclitaxel. AR antagonists have been used in combination with conventional treatments such as radiotherapy and chemotherapy with satisfactory results (24, 48, 49). Given the evidence reviewed above, the combination of AR-targeted therapy with other therapies may improve the efficacy of BC therapy, and, therefore, its clinical effectiveness needs to be further explored. Apart from adjuvant therapy, there are few studies on ARs in neoadjuvant therapy, which may be an intriguing topic.

The keyword “men” in the top 25 keywords with the strongest citation bursts grabbed our attention. For male breast cancer, the role of ARs also requires further study. We know that estrogen in men is mainly derived from androgens, so aromatase inhibitors targeting androgens are pivotal in the treatment of male breast cancer. Aromatase inhibitors are not used alone; they need to be used with other drugs that inhibit androgen production by the testis, so the effect of combining androgen receptor inhibitors with other drugs is also worth exploring (50). Similarly, the androgen receptor plays a significant role in the process of androgen action; however, its therapeutic value in male breast cancer seems to be poorly studied. In a recent meta-analysis, the use of SRD5A inhibitors was assessed as a possible treatment for male BC to reduce androgen levels, as this treatment has been used to treat benign prostatic hyperplasia and androgenetic alopecia. Unfortunately, this study did not find promising results (25). This provides a new direction for future research. Likewise, another keyword, “tumor-infiltrating lymphocyte”, may be an indication of AR’s potential mechanism on BC’s prognosis. Much work is still needed to further explore their relationship.

There are some limitations to our study. First, due to the limitations of scientometric software, it is difficult to merge the results of two databases directly, so we only searched on the Web of Science without combining the results with those from other search databases such as Embase, PubMed, or Scopus. The Web of Science does not cover all journals in any discipline. Moreover, it has a more focus on English journals, leading to a lack of non-English language journals’ representation. Still, the Web of Science is the most used literature database for scientometric studies. With some functions for primary analysis like “analyze results” and “create citation report”, it is the friendliest and the easiest tool to use. Most bibliometric software could identify the format from the Web of Science. It is not possible to get the complete information of an article, for example, the author’s country of affiliation. However, it can be addressed by manually searching for the author (51). The criteria for screening articles are somewhat subjective, thus relevant criteria or comprehensive opinions of multiple experts for screening articles are required. The applications we used to analyze the result also have some shortages. For example, VOSviewer’s co-citation analysis of cited authors from the Web of Science data includes only the first author of a cited document, while other authors are not considered. Furthermore, the most-cited articles may be multidisciplinary and cover a wide range, resulting in low specificity for our research field. These articles’ publication dates may be ages ago and without having been updated in time, so their conclusions may not be consistent with recent findings, which weakens their citation value. As the publication published this year was not included in the analysis, our study is only applicable to the time points of the current study conducted (October 3, 2021). However, we believe that our study can provide some future research ideas for researchers in relevant fields.

## Conclusion

The role of estrogen and progesterone receptor in the development of breast cancer has been recognized worldwide. In recent years, many researchers have begun to explore the role and value of androgen receptors in breast cancer. This scientometric study analyzed the information from relevant articles from 2011 to 2020 on the value of androgen receptors in breast cancer, revealing the collaboration networks of contributing countries, institutions, journals, and authors, and providing the research results of landmark articles, as well as the research hotspots and directions to be studied. In the past decades, related research in this area continued to deepen, transitioning from molecular mechanism level to clinical application level. This study provides guidance for relevant researchers to develop a new targeted drug or even more, combine androgen receptor-targeted therapy with other treatments in the future. We can infer that the emerging themes such as “androgen receptor expression”, “growth-factor receptor”, “neoadjuvant chemotherapy”, and “tumor-infiltrating lymphocyte” need further investigation. The hotspots, including “molecular subtype”, “peripheral blood”, “pathological complete response”, “tumor-infiltrating lymphocyte”, “men”, “migration”, and “small molecular inhibitor” are worth exploring deeper. The optimal threshold of ARs and its application in male breast cancer are also interesting topics deserving exploration. Maybe the upstream pathways of androgen receptors can explain the discrepancy of its prognostic value in breast cancer. In view of today’s heavy cancer burden, attention to androgen receptors will continue to increase, which will lead to advances in clinical treatment modalities.

## Data Availability Statement

The original contributions presented in the study are included in the article/**Supplementary Material**, further inquiries can be directed to the corresponding author/s.

## Author Contributions

YXC, ZL and JiZ contributed to conception and design of the study. All authors contributed to the development of the protocol. YL and JHC provided guidance on the use of these analyzing applications and statistical analysis. LC extracted the dataset from the Web of Science, performed the statistical analysis, and was a major contributor to writing the manuscript. JHC, ZJ, JuZ, YKC, JW, and DZ were involved in the interpretation of the study findings. JiZ, YL, and JHC revised the article critically. All authors read and gave final approval for the submitted versions.

## Funding

This work was supported by the Special Fund Project of Guangdong Science and Technology (210728156901524, 210728156901519), Medical Scientific Research Foundation of Guangdong Province, China (grant number A2021432, B2021448), Shantou Medical Science and Technology Planning Project (grant number 210521236491457, 210625106490696), and the Undergraduate Innovation Training Project of Shantou University (grant number 31/38/47/54).

## Conflict of Interest

The authors declare that the research was conducted in the absence of any commercial or financial relationships that could be construed as a potential conflict of interest.

## Publisher’s Note

All claims expressed in this article are solely those of the authors and do not necessarily represent those of their affiliated organizations, or those of the publisher, the editors and the reviewers. Any product that may be evaluated in this article, or claim that may be made by its manufacturer, is not guaranteed or endorsed by the publisher.

## Glossary

**Table:**

AR	androgen receptors
BC	breast cancer
AIs	aromatase inhibitors
SERMs	selected estrogen receptor modulators
TNBCs	triple-negative breast cancers
HER2	human epidermal growth factor receptor 2
WoS	Web of Science
SCI-EXPANDED	Science Citation Index Expanded
SSCI	Social Sciences Citation Index
AHCI	Arts & Humanities Citation Index
ESCI	Emerging Sources Citation Index
CCR-EXPANDED	Current Chemical Reactions
IC	Index Chemicus
Mesh	Medical subject headings
ACI	average citations per term
h-index	Hirsch index
LAR	luminal androgen receptor
FOXA1	Forkhead box A1
BL1	basal-like 1
BL2	basal-like 2
IM	immunomodulatory
M	mesenchymal
MSL	mesenchymal stem-like
ROC	receiver operating characteristic
IHC	immunohistochemistry
CTCs	circulating tumor cells
PARP	poly (ADP-ribose) polymerase
CDK4/6	cyclin-dependent kinases 4 and 6
PI3K	phosphatidylinositol-3-kinase
SRD5A	steroid 5α-reductase
