# Supplementary material for: Global Trends in Research of Androgen Receptor Associated With Breast Cancer From 2011 to 2020: A Scientometric Analysis
Source: Front Endocrinol (Lausanne). 2022 Jun 21;13:887612. doi: 10.3389/fendo.2022.887612 (PMC9253269; doi:10.3389/fendo.2022.887612)
Supplement: Supplementary file 1 [file DataSheet_1.docx]

Supplementary Material

**Supplementary Table S1** Top 10-cited documents in the research scope of androgen receptor and breast cancer with corresponding authors, publication year, journal, volume, issue, page, total citations and total citations per year.

| Authors* | Article | Journal | Year | Vol | Issue | Page | TC | TC per year |
| --- | --- | --- | --- | --- | --- | --- | --- | --- |
| Lehmann, Brian D.; Bauer, Joshua A.; Chen, Xi; Pietenpol, Jennifer A. | Identification of human triple-negative breast cancer subtypes and preclinical models for selection of targeted therapies | JOURNAL OF CLINICAL INVESTIGATION | 2011 | 121 | 7 | 2750-2767 | 2766 | 251.45 |
| Bianchini, Giampaolo; Balko, Justin M.; Mayer, Ingrid A.; Gianni, Luca | Triple-negative breast cancer: challenges and opportunities of a heterogeneous disease | NATURE REVIEWS CLINICAL ONCOLOGY | 2016 | 13 | 11 | 674-690 | 926 | 154.33 |
| Zaret, Kenneth S.; Carroll, Jason S. | Pioneer transcription factors: establishing competence for gene expression | GENES & DEVELOPMENT | 2011 | 25 | 21 | 2227-2241 | 924 | 84 |
| Musgrove, Elizabeth A.; Caldon, C. Elizabeth; Barraclough, Jane; Sutherland, Robert L. | Cyclin D as a therapeutic target in cancer | NATURE REVIEWS CANCER | 2011 | 11 | 8 | 558-572 | 872 | 79.27 |
| Yang, Yanzhong; Bedford, Mark T. | Protein arginine methyltransferases and cancer | NATURE REVIEWS CANCER | 2013 | 13 | 1 | 37-50 | 544 | 60.44 |
| Hudis, Clifford A.; Gianni, Luca | Triple-Negative Breast Cancer: An Unmet Medical Need | ONCOLOGIST | 2011 | 16 |  | 1-11 | 505 | 45.91 |
| Burstein, Matthew D.; Tsimelzon, Anna; Poage, Graham M.; Brown, Powel H. | Comprehensive Genomic Analysis Identifies Novel Subtypes and Targets of Triple-Negative Breast Cancer | CLINICAL CANCER RESEARCH | 2015 | 21 | 7 | 1688-1698 | 498 | 71.14 |
| Lai, Ashton C.; Crews, Craig M. | Induced protein degradation: an emerging drug discovery paradigm | NATURE REVIEWS DRUG DISCOVERY | 2017 | 16 | 2 | 101-114 | 471 | 94.2 |
| Zhou, Hongyu; Beevers, Christopher S.; Huang, Shile | The Targets of Curcumin | CURRENT DRUG TARGETS | 2011 | 12 | 3 | 332-347 | 430 | 39.09 |
| Issa, Fatiah; Kassiou, Michael; Rendina, Louis M. | Boron in Drug Discovery: Carboranes as Unique Pharmacophores in Biologically Active Compounds | CHEMICAL REVIEWS | 2011 | 111 | 9 | 5701-5722 | 426 | 38.73 |

* First, second, third, and last authors

**Supplementary Table S2** Top 10 popular journals and cited journals. A) Top 10 popular journals regarding the number of related articles. B) Top 10 popular journals regarding the number of citations.

1. Top 10 popular journals

| Popular journals | Articles | 2022 impact factor | 2022 JCR partition |
| --- | --- | --- | --- |
| ONCOTARGET | 72 | -- | -- |
| PLOS ONE | 63 | 3.24 | Q3 |
| BREAST CANCER RESEARCH AND TREATMENT | 50 | 4.87 | Q2 |
| ENDOCRINE-RELATED CANCER | 44 | 5.68 | Q2 |
| CANCERS | 40 | 6.64 | Q2 |
| CLINICAL CANCER RESEARCH | 40 | 12.53 | Q1 |
| BMC CANCER | 35 | 4.43 | Q3 |
| JOURNAL OF STEROID BIOCHEMISTRY AND MOLECULAR BIOLOGY | 35 | 4.29 | Q2 |
| ONCOGENE | 33 | 9.87 | Q1 |
| MOLECULAR AND CELLULAR ENDOCRINOLOGY | 32 | 4.10 | Q3 |

1. Top 10 cited journals.

| Cited journals | Citations | 2022 impact factor | 2022 JCR partition |
| --- | --- | --- | --- |
| CLINICAL CANCER RESEARCH | 3077 | 12.53 | Q1 |
| Journal of Clinical Investigation | 3067 | 14.81 | Q1 |
| Nature Reviews Cancer | 2464 | 60.72 | Q1 |
| ONCOGENE | 2087 | 9.87 | Q1 |
| ONCOTARGET | 1841 | -- | -- |
| PLOS ONE | 1725 | 3.24 | Q3 |
| PROCEEDINGS OF THE NATIONAL ACADEMY OF SCIENCES OF THE UNITED STATES OF AMERICA | 1358 | 11.21 | Q1 |
| ENDOCRINE-RELATED CANCER | 1324 | 5.68 | Q2 |
| BREAST CANCER RESEARCH AND TREATMENT | 1275 | 4.87 | Q2 |
| BREAST CANCER RESEARCH | 1078 | 6.47 | Q2 |


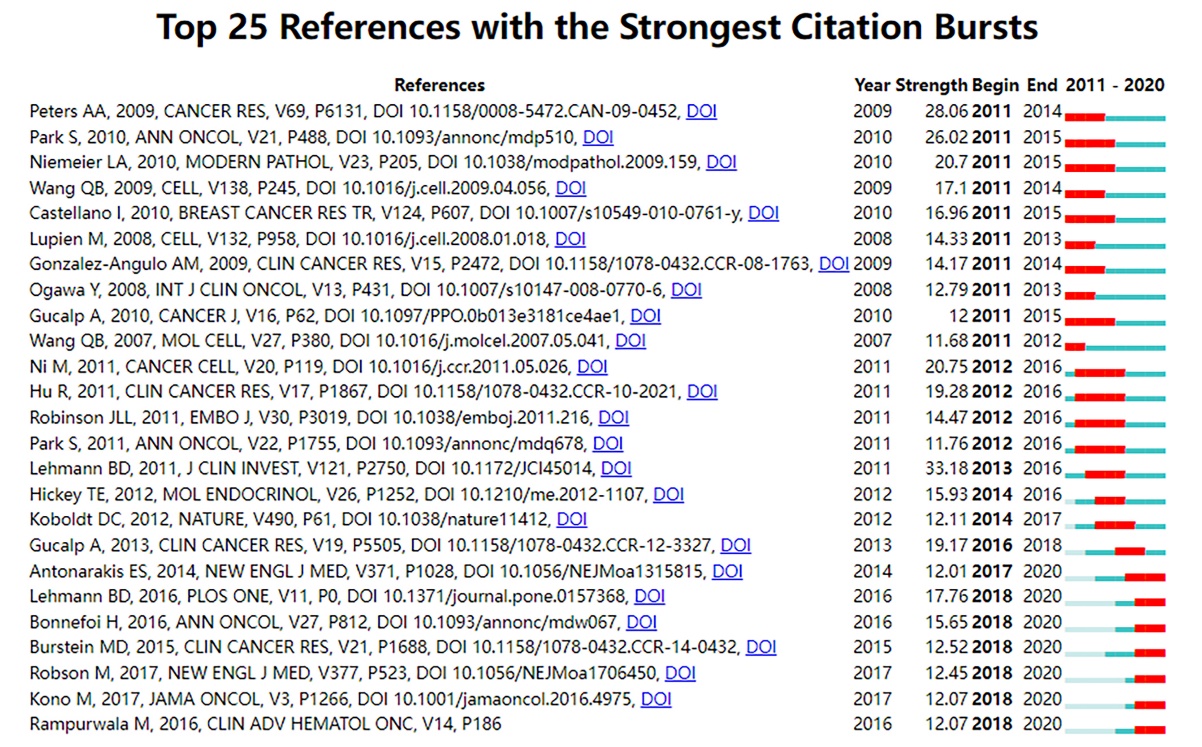


**Supplementary Figure S1** Top 25 references with the strongest citations burst.


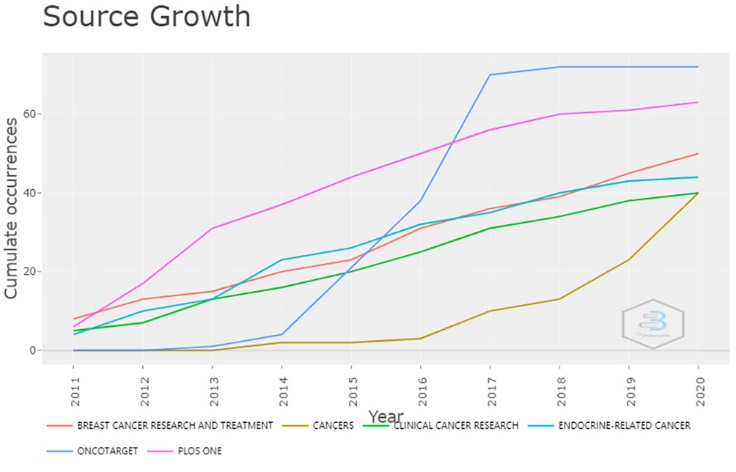


**Supplementary Figure S2** Source growth over the years related to this research scope.


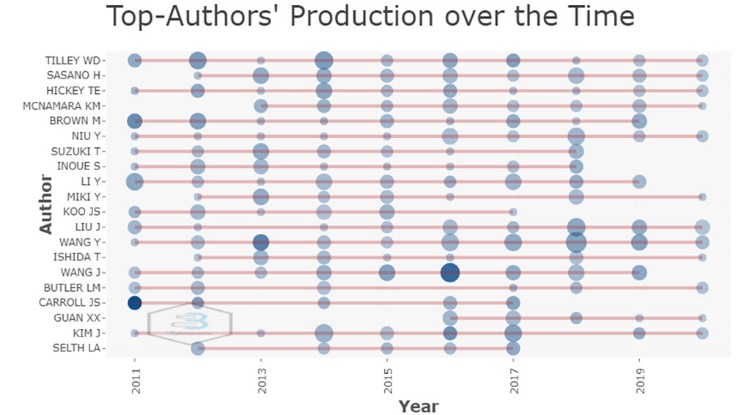


**Supplementary Figure S3** Authors’ production of this field over time.


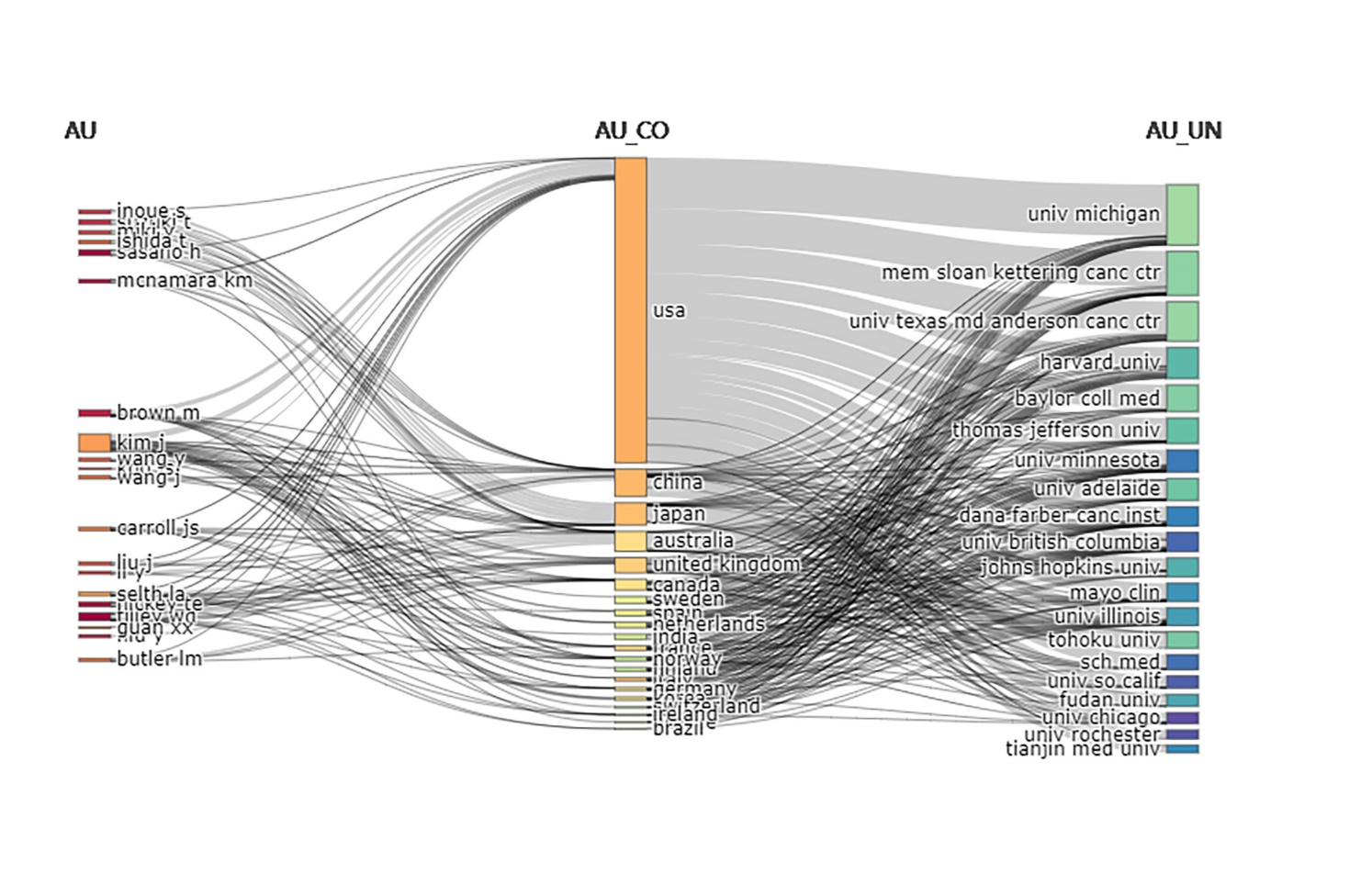


**Supplementary Figure S4** Three-field plot among institutions(right), the most productive countries(middle) and top authors(left).

**
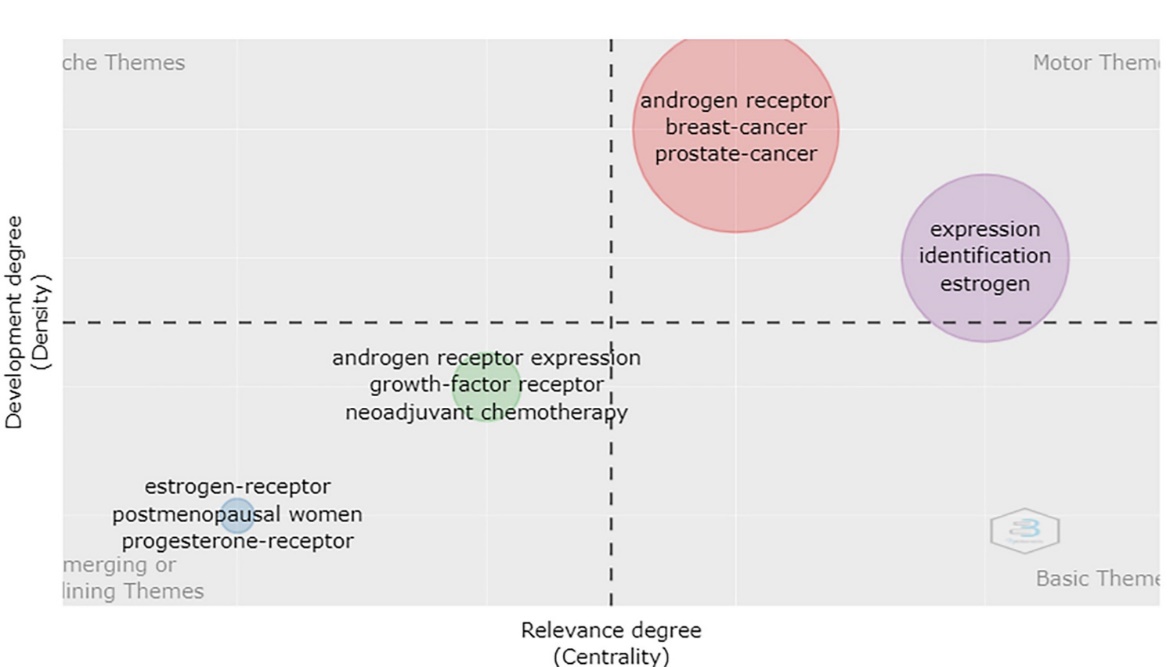
**

**Supplementary Figure S5** Keywords plus of four quadrants. (a) motor themes (first quadrant); (b) highly developed and isolated themes (second quadrant); (c) emerging or declining themes (third quadrant); (d) basic themes (fourth quadrant).


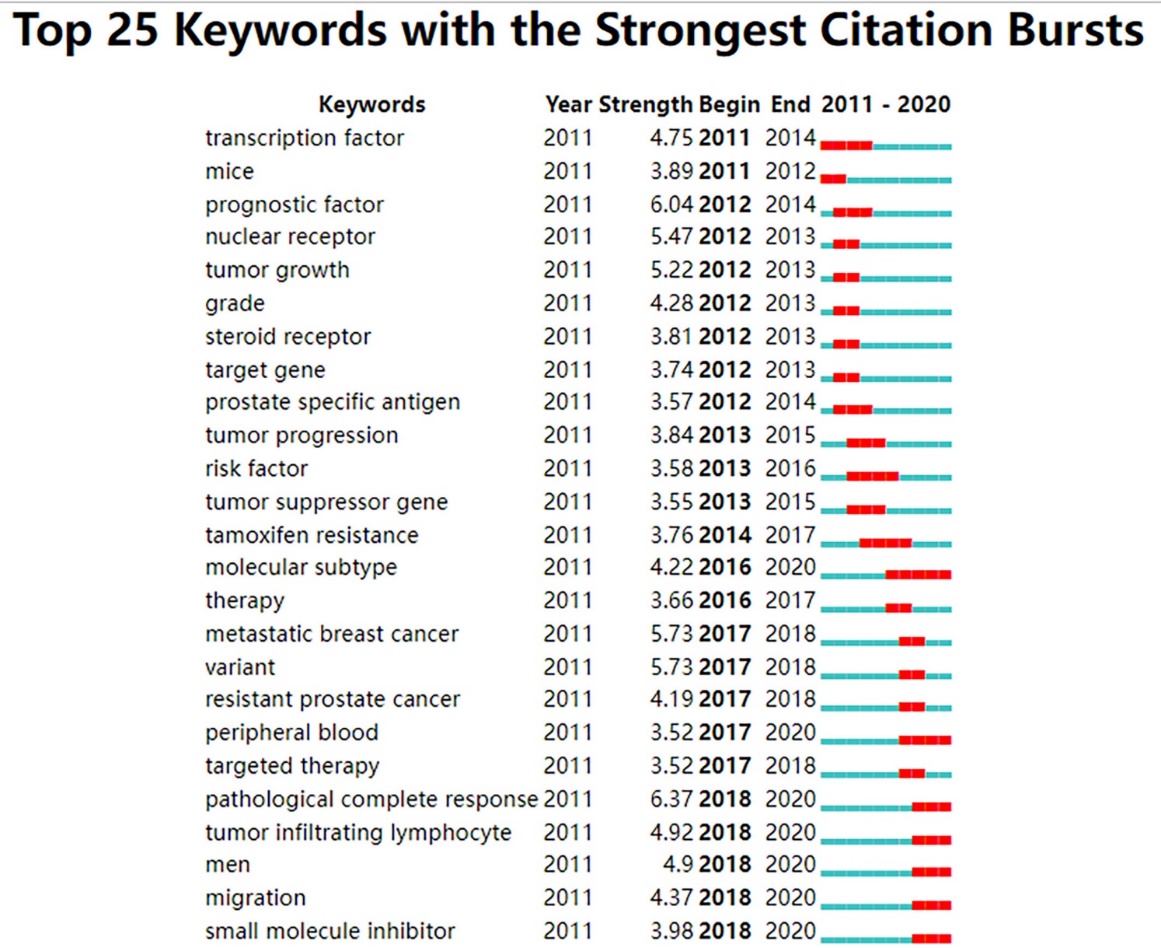


**Supplementary Figure S6** Top 25 keywords with the strongest citations bursts of this field.


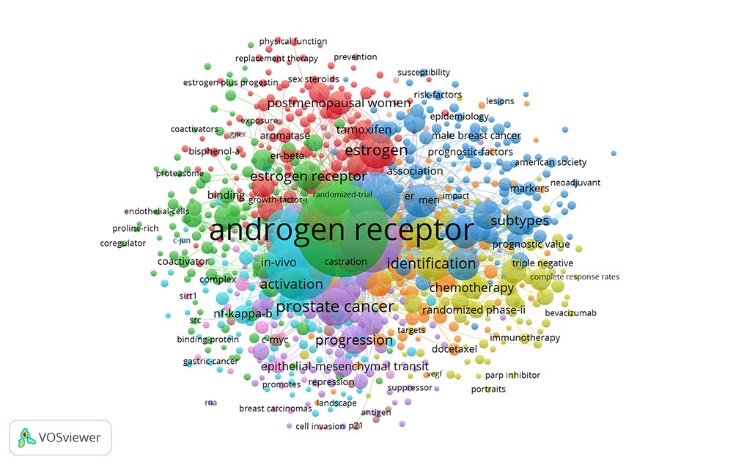

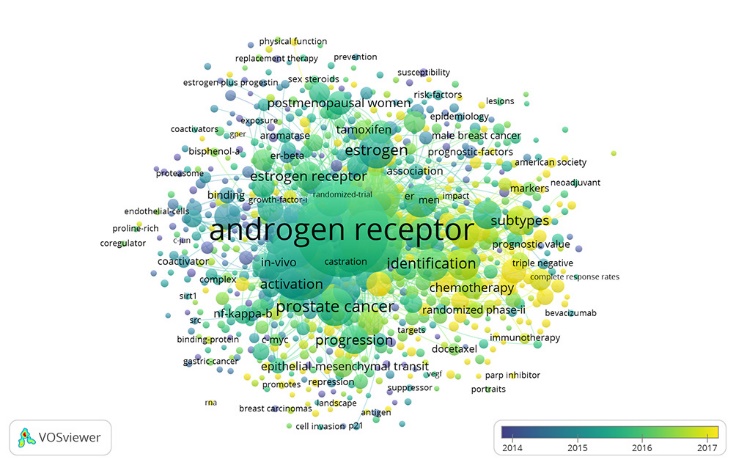


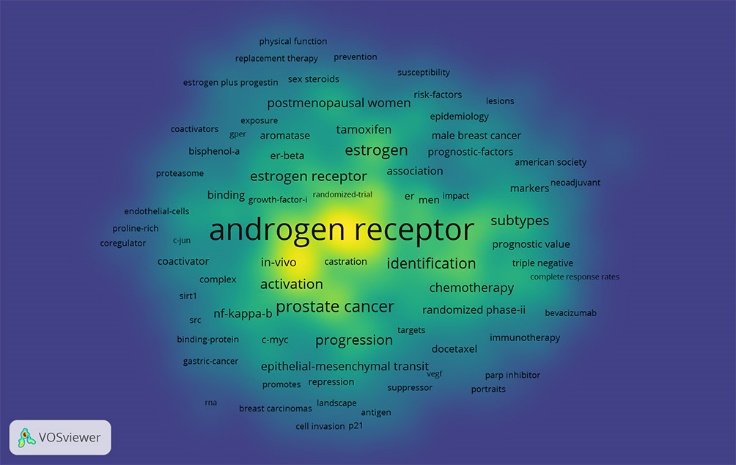


**Supplementary Figure S7** Co-occurrence analysis of keywords. (A) Network mapping of keywords of related studies (10 clusters). (B) Overlay mapping of keywords according to average publication year (blue means earlier, yellow means later). (C) Density mapping of keywords according to the frequency of appearance (yellow means more frequent).
