# Supplementary material for: Global Trends in Research of Androgen Receptor Associated With Breast Cancer From 2011 to 2020: A Scientometric Analysis
Source: Front Endocrinol (Lausanne). 2022 Jun 21;13:887612. doi: 10.3389/fendo.2022.887612 (PMC9253269; doi:10.3389/fendo.2022.887612)
Supplement: Supplementary file 2 [file Table_3.docx]

Supplementary Material

**Supplementary Table S3** Top 5 authors with the most times cited of their publications in the research scope of androgen receptor and breast cancer.

| Author | Documents | Citations | Total link strength |
| --- | --- | --- | --- |
| Sanders ME | 4 | 3879 | 1527 |
| Lehman BD | 6 | 3495 | 1852 |
| Pietenpol JA | 6 | 3495 | 1852 |
| Chen X | 4 | 3025 | 1375 |
| Bauer JA | 2 | 2929 | 1319 |
| Shyr Y | 2 | 2790 | 1215 |
